# “We want to live a little longer and our family want[s] us around”: A summative content analysis of adherence to COVID‐19‐related guidelines using the Theoretical Domains Framework

**DOI:** 10.1111/bjhp.12591

**Published:** 2022-03-23

**Authors:** Jessica Z. Leather, Chris Keyworth, Tracy Epton, Joanna Goldthorpe, Fiona Ulph, Christopher J. Armitage

**Affiliations:** ^1^ Division of Psychology and Mental Health Manchester Centre for Health Psychology School of Health Sciences The University of Manchester UK; ^2^ NIHR Greater Manchester Patient Safety Translational Research Centre The University of Manchester Manchester Academic Health Science Centre UK; ^3^ School of Psychology University of Leeds UK; ^4^ Manchester University NHS Foundation Trust Manchester Academic Health Science Centre UK

**Keywords:** COVID‐19, Theoretical Domains Framework, summative content analysis, adherence, government guidance

## Abstract

**Objective:**

Public adherence to COVID‐19‐related government guidance varied during the initial lockdown in the UK, but the determinants of public adherence to such guidance are unclear. We capture spontaneous reflections on adherence to UK government guidance from a representative UK sample, and use the TDF to identify key determinants of COVID‐related behaviours.

**Design:**

The design was cross‐sectional.

**Methods:**

Qualitative data were collected from a large sample of UK adults (*N = *2,252) via an online questionnaire as part of a wider survey about the UK public’s responses to the government’s COVID‐19‐related guidance. Summative content analysis was used to identify key guideline terms in the data, followed by latent analysis to interpret the underlying meanings behind the terms using the TDF as an analytical framework.

**Results:**

Six TDF domains were identified in the data: Environmental Context and Resources; Beliefs about Consequences; Social Influences; Memory, Attention and Decision Processes; Emotion; and Knowledge. Although the samples were motivated and capable of adhering, limitations in their environments, resources, and social support mechanisms restricted behaviour. Self‐reported adherence was sensitive to positive and negative beliefs about the effectiveness of the measures, in addition to interpretations of the terms ‘essential’ and ‘necessary’ in the guidance.

**Conclusions:**

Despite extensive structural obstacles to adherence, the majority of the British public were able to follow government COVID‐19‐related instructions, provided they had sufficient resources, social support, and positive perceptions about the effectiveness of the measures. Ambiguities surrounding key terminology in the guidance left room for interpretation, which may have contributed to non‐adherence.

**Statement of contribution**

***What is already known on this subject?***

- Behavioural measures were implemented in worldwide lockdowns to suppress the spread of COVID‐19.
- Public adherence differed between stay home, hand hygiene, and physical distancing measures.
- Discrete drivers of adherence have been identified, but comprehensive frameworks were not applied.

***What does this study add?***

- Local resources and community support are needed for sustained adherence to behavioural measures.
- Knowledge provision about COVID‐19 must be trustworthy and unambiguous to avoid misinterpretation.
- Interventions to bolster well‐being and morale could benefit people under behavioural restrictions.

## Background

Since the COVID‐19 pandemic was declared in March 2020 (World Health Organization, 2020), governments and public health bodies implemented behavioural advice and ‘lockdown’ measures to control the spread of the virus (Coroiu, Moran, Campbell, & Geller, 2020; Pak, McBryde, & Adegboye, 2021). The UK government advised specific health measures to ‘Stay Home, Protect The NHS, Save Lives’: this involved maintaining hand hygiene by washing hands with soap and water for 20 seconds; physical distancing (remaining 1–2 metres apart from anybody not living in one’s household; Sørensen, Okan, Kondilis, & Levin‐Zamir, 2021); and announcing a national ‘stay‐at‐home’ order to curb non‐essential travel and gatherings (Public Health England, 2020). Imposing measures to enforce the 1–2 metre physical distancing rule reduced virus transmission in the UK (Jarvis et al., 2020) and worldwide (Islam, Vidot, & Camacho‐Rivera, 2021; McGrail, Dai, McAndrews, & Kalluri, 2020), leading to a gradual easing of restrictions (Han et al., 2020) and reduced mortality (Margraf, Brailovskaia, & Schneider, 2021). However, further lockdowns were implemented in the UK to control new variants and increased infection rates (Kirby, 2021; Merchant, Kow, & Hasan, 2021), and it is likely that similar lockdown measures could be needed in the future to control COVID‐19, other novel coronaviruses, or other anticipated pandemics (Thoradeniya & Jayasinghe, 2021). It is therefore important to learn as much as possible from these early experiences.

The success of preventative measures relies upon sustained adherence by members of the public (Chater et al., 2021; Michie et al., 2020; Speight, Skinner, Hately‐Browne, & Abraham, 2020). Adherence to the first cluster of government guidance was initially high in the UK (Armitage, Keyworth, Leather, Byrne‐Davis, & Epton, 2021), China (Gao et al., 2020), the USA (Qeadan et al., 2020), and Western European countries (Margraf, Brailovskaia, & Schneider, 2020). However, further inspection of the data suggests that adherence differs between different guideline behaviours. For example, a survey collected the week before the initial UK lockdown from a sample of 2,108 adults suggested 86% washed their hands more frequently, but only 45% avoided crowded places and social events (Atchison et al., 2021). Similarly, among a representative sample of 11,342 working‐age Japanese citizens, 86% adhered to hand hygiene measures during the initial lockdown phase, while 57% adhered to physical distancing measures (Muto, Yamamoto, Nagasu, Tanaka, & Wada, 2020). Data collected during May 2020 (after the partial easing of UK lockdown restrictions) from a sample of 681 people in North London found that 90% of the sample could not maintain 2‐metre distance from other people, when outside for permitted reasons (Hills & Eraso, 2021). This inconsistency has prompted researchers to examine why people do, or do not, adhere to government instructions.

A number of studies have utilized quantitative questionnaire data to identify potentially modifiable determinants of guideline adherence, such as attitudes towards measures (Czeisler et al., 2021; Gao et al., 2020); pro‐social motivations about ‘civic duty’ or ‘social responsibility’ to protect others (Coroiu et al., 2020; Gouin et al., 2021); and beliefs surrounding risk and susceptibility (Xie, Liang, Dulebenets, & Mei, 2020). Structural barriers to physical distancing have been identified, such as environmental restrictions in houses of multiple occupancy (Hills & Eraso, 2021), and caring responsibilities (Keyworth, Epton, Byrne‐Davis, Leather, & Armitage, 2021). However, these survey studies rely on direct questioning and survey methods (Mieth, Mayer, Hoffmann, Buchner, & Bell, 2021), so there is little understanding about what is meant when members of the public say they are adhering to the government’s instruction. Binary yes/no responses and numerical ratings of adherence provide limited insight into the ways people interpret and act upon the broad terminology used in health guidance, particularly where several complex behaviours are involved; the analysis of spontaneous qualitative responses may capture some of the nuances missed by existing research, and identify opportunities to improve future interventions and public health messages (Braun, Clarke, Boulton, Davey, & Mcevoy, 2020).

Interviews and focus groups have been used to investigate participant‐generated determinants of adherence among a Canadian sample (Benham et al., 2021); experiences of complying with ‘stay‐at‐home’ measures in the UK (Williams, Armitage, Tampe, & Dienes, 2020); and adherence within a UK Muslim community (Hassan, Ring, Tahir, & Gabbay, 2021). However, the above quantitative and qualitative evidence is limited by a lack of theoretical grounding to guide the identification of salient determinants. To address this deficit, some models such as the Theory of Planned Behaviour (Margraf et al., 2020; Sturman, Auton, & Thacker, 2020), Health Action Process Approach (Beeckman et al., 2020), and Extended Parallel Process Model (Lithopoulos, Liu, Zhang, & Rhodes, 2021) have been used to explore the extent that attitudes, risk perception, and self‐efficacy predict adherence to COVID‐19 guideline behaviours. Positive attitudes and knowledge of the guidelines predicted intentions to adhere to COVID‐19 measures in accordance with the Theory of Planned Behaviour (Sturman et al., 2020). Perceived capability, measured as self‐efficacy, was a strong predictor of intentions to adhere across the Health Action Process Approach and Extended Parallel Process Models, while negative well‐being, lack of social support, and beliefs about the exaggeration of COVID‐19 were associated with barriers to adherence (Beeckman et al., 2020; Lithopoulos et al., 2021). Although these studies have a theoretical basis and make feasible recommendations to target amenable factors, such as perceived threat, efficacy, and attitudes, a disadvantage is that the models they are based upon are not comprehensive, so do not offer a full range of potential strategies for change to remediate low adherence.

A solution to the limitations of existing research is to utilize the Theoretical Domains Framework (TDF) (Atkins et al., 2017; Cane, O’Connor, & Michie, 2012) to explore adherence. The TDF integrates several theories of behaviour change into a single framework of behavioural determinants, which is advantageous because it offers a single, comprehensive tool for analysis instead of numerous overlapping models. This framework comprises fourteen domains encapsulating cognitive (e.g. Intentions), affective (e.g. Emotions), social (e.g. Social Influences), and environmental (e.g. Environmental Context and Resources) influences on behaviour, which can be used to categorize the determinants of behaviour. Although the TDF was developed for implementation research to understand and change healthcare professional behaviour (Cane et al., 2012), it has been applied to complex health behaviours by members of the public, such as physical activity (Haith‐Cooper, Waskett, Montague, & Horne, 2018), medication adherence (Prajapati et al., 2019), and use of sexual health services (Cassidy et al., 2018). The TDF is part of the Behaviour Change Wheel for intervention development (Michie, Atkins, & West, 2014), meaning there is potential to use salient domains to select candidate intervention functions, behaviour change techniques, and policy categories to form behaviour change interventions (Cane, Richardson, Johnston, Ladha, & Michie, 2015). Therefore, the TDF is an appropriate framework to understand reflections on adherence to COVID‐19‐related guidance, and holds the potential to identify potentially modifiable targets for behaviour change at the individual, community, and policy level.

### Aims

The present study aimed to: (a) Capture spontaneous reflections on adherence to UK government guidance from a representative UK sample, and (b) Use the TDF to identify key determinants of COVID‐related behaviours.

## Methods

### Design and procedure

The design was cross‐sectional. Qualitative data were collected from a large sample of UK adults (*N = *2,252) designed to be representative of the UK population via an online questionnaire as part of a wider survey that assessed the UK public’s adherence to the government’s COVID‐19‐related guidance, and identified prevalent challenges to adherence (Armitage et al., 2021; Keyworth et al., 2021). Ethical approval was obtained from a University Research Ethics Committee (Ref: 2020‐9551‐15105) and participants gave informed consent at the beginning of the survey. The survey was conducted through a survey panel company in April 2020 (YouGov). The survey company aimed to recruit a sample representative of the UK population from their participant pool using quotas for age, ethnicity, gender, and country of residence. Participants were incentivized to take part with a points‐based system, where respondents accumulate points for completing surveys in exchange for prize draws or cash payment. Survey responses were collected and anonymized by the company, then transferred to the researchers for analysis.

### Measures

Sociodemographic factors, such as age, gender, ethnicity, and social grade were collected, in addition to country of residence, work status, marital status, and information about children. Participants were provided with a single questionnaire item: ‘What challenges, if any, are you facing in following the UK government’s coronavirus guidance?’. An open‐ended text field captured their responses to allow for spontaneous descriptions of adherence, providing the potential to capture a diverse range of perspectives, in addition to rich, focused accounts of adherence‐related behaviours (Braun et al., 2020). Since the survey question was not structured around TDF domains, respondents could naturally report on determinants of their behaviour. This is advantageous because it expands on the quantitative measures of adherence captured in the wider survey (Keyworth et al., 2021), and is not limited to barriers and enablers conceptualized by the TDF, whilst using the TDF as a tool to organize the data into *a priori* themes (McGowan, Powell, & French, 2020).

### Analysis

Summative content analysis was used to analyse the data, to focus the analysis around key words derived from the government guidance (Hsieh & Shannon, 2005). This is an analytical approach that begins with the identification of key terms in a dataset, followed by a latent analysis to interpret the underlying meanings behind the terms (Holsti, 1969; Morse & Field, 1995). Summative content analysis was selected as an appropriate exploratory method after data collection was completed because it offered a strategy to quantify and compare the prominence of key phrases derived from government instructions, and interpret and reflect upon the ways that members of the public understood the instructions.

#### Stage 1: Identification of reflective responses

A search strategy was developed by the research team to capture the key terminology used in the government guidelines (e.g. ‘wash’, ‘stay’, ‘distanc*’) (Appendix A), and terms associated with reasons for or against adherence to the guidelines (e.g. ‘have to’, ‘rarely’, ‘because’) (Appendix B). Guideline terms were selected from the ‘Stay Home, Protect The NHS, Save Lives’ campaign; this was active during data collection and aimed at everyone in the UK (Public Health England, 2020). The search strategies were executed in Microsoft Excel to identify responses containing reflections on adherence to government instructions; the results of both searches were combined, and duplicates eliminated.

#### Stage 2: Latent analysis

Latent coding analysis was used to interpret reflections on adherence to government‐related instructions from data identified in Stage 1. This involved selecting statements that provided any reasons for adherence or non‐adherence to any of the guideline measures, by hand‐searching the responses. Two members of the research team (JZL and CK) analysed the data independently, and coding discrepancies were resolved through discussion until an agreement was reached.

#### Stage 3: Framework analysis using the TDF

Microsoft Excel was used to facilitate the coding and organization of themes for analysis; a framework approach (Gale, Heath, Cameron, Rashid, & Redwood, 2013) was used by one coder (JZL) to map the data onto relevant domains of the TDF to explore both predetermined and emergent themes. This allowed the coder to identify constructs that may be amenable to change using deductive (first level) coding (Atkins et al., 2017). A sample of 50 responses was checked by both coders (JZL and CK) to check JZL’s consistency and ensure inter‐coder reliability (O’Connor & Joffe, 2020); both coders agreed on 100% of the codes from this sample. 17 further responses were queried by JZL for not fitting any domains, which were then categorized into appropriate TDF domains following discussion with CK (this process is detailed in Appendix C). Some responses mentioned numerous determinants that fit more than one domain; these quotes were mapped in their entirety to relevant domains. Finally, barriers and enablers within each domain were coded inductively (second level) using a priori themes. Since the COM‐B model characterizes behaviour as a result of an interaction between capability, opportunity, and motivation (Michie et al., 2014), overlapping themes across TDF domains were anticipated; these were identified and labelled during second‐level coding (an example of this is illustrated in Appendix D).

## Results

### Descriptive analysis

Demographic information can be found in Appendix E. Participants had a mean age of 50.34 years (*SD* = 17.02) and 1,234 (54.8%) were women. 2,095 (93.0%) were White; 34 (1.5%) were from mixed/multiple ethnic groups, 54 (2.4%) were Asian, 16 (0.7%) were Black, and 11 (0.5%) were from other ethnic groups. In terms of social grade, 1,294 (57.0%) worked in non‐manual occupations (NRS grades A‐C1), and the remainder were unemployed or working in manual occupations (NRS grades C2‐E). 618 (27.4%) of the sample were retired. Almost half of the sample were married or partnered (*N = *1,089, 48.6%); and 1,333 (59.2%) were parents.

One hundred and seventy‐one participants provided a null response to the open‐ended questionnaire item. Such responses consisted of blanks, punctuation marks, expletives, emoji, key smashes, variations on abbreviations such as ‘N/A’ or ‘DK’, and single words unrelated to the guidance such as “*Excellent*” (Participant 35). Null responders were younger on average (M = 44.1, SD=16.4) than the wider sample. A greater proportion were men (*N = *98, 57.6%); aged between 18–34 (*N = *59, 34.5%); and were of a lower social grade (*N = *73, 42.7%).

After applying Stage 1 of the search strategy, 1,695 responses (75.3%) included at least one government guideline‐related term. Captured guideline terms (Public Health England, 2020) can be found in Table 1. Staying at home was mentioned most frequently in 1,198 (53.2%) unique statements. Hand washing (*N = *717 statements; 31.8%) and physical distancing (*N = *669 statements; 29.7%) were mentioned less. There were few demographic differences between those who mentioned different behaviours. Participants who mentioned hand washing were older (M = 51.13, SD=16.59) than those who mentioned physical distancing (M = 49.83, SD=16.45) or staying at home (M = 49.55, SD=17.32), and a greater proportion of women mentioned hand washing (*N = *449, 62.2%) than staying at home (*N = *725, 60.5%) or physical distancing (*N = *383 57.2%).

**Table 1:** Use of key guideline terms in 2,081 statements

Category	Terms	Mentions	Statements
Guideline‐related			
Hand	"Wash*" (washing)	762	697
Washing	"Sanit*" (sanitise, sanitary, sanitiser)	84	77
Staying at home	"Stay*" (staying)	663	624
	"Shield" (shielding)	39	36
	"Home"	851	709
	"House"	422	348
	“Isolat*” (isolating, self‐isolating, isolation)	122	120
Physical distancing	"Distanc*" (distance, distanced, distancing)	581	557
	"Metre*" (2 Metres)	102	99
	“2m”	94	91
	"Gather" (gathering, gatherings)	4	4
Guideline exceptions/ exemptions	"Essential" (essentials)	380	366
	"Necess*" (necessary, necessity, necessities)	106	105
	“Exercis*” (exercise, exercising)	369	352
	"Work*" (working, "key worker")	532	399
Adherence‐related			
Modals	"Must"	4	4
	“Can*”	137	120
	“Have”	483	336
	“Need”	89	75
	“Won*”	4	4
	“Will”	47	39
	“Should”	13	12
	“Shall”	2	2
Frequency	“Rare*”	24	20
	“Regular*”	123	118
	“Occasion*”	50	49
	"Always"	38	34
	"Never"	19	18
Reflective terms	“Because”; “just”; “only”; “forg*”; “intent*”; “unless”; “other than”; “except”; “however”; “although”; “instead”	1,188	1,083

In terms of adherence‐related terms, most participants used reflective terminology (*N = *1,083; 48.1%) (e.g. ‘*I try to keep to all the instructions… because I don't want to catch the virus or transmit it’*; Participant 1076). Modals related to adherence were used less often (*N = *592; 26.3%) (e.g. ‘… *only shopping when I need to’*; Participant 827); however, frequency terminology was used least often (*N = *239; 10.6%) (e.g. ‘… *not washing my hands any more regularly than before’*; Participant 949).

Of the 1,695 responses containing a guideline‐related term, 1,098 (48.8%) also included an adherence‐related term, making them eligible for latent analysis. The remaining statements were hand‐searched for any responses that contained salient reflections about adhering to government instructions. A further 113 responses were included, meaning a total of 1,211 (53.8%) statements were selected for latent analysis.

### Latent analysis: reflections on adherence

**Figure 1:**
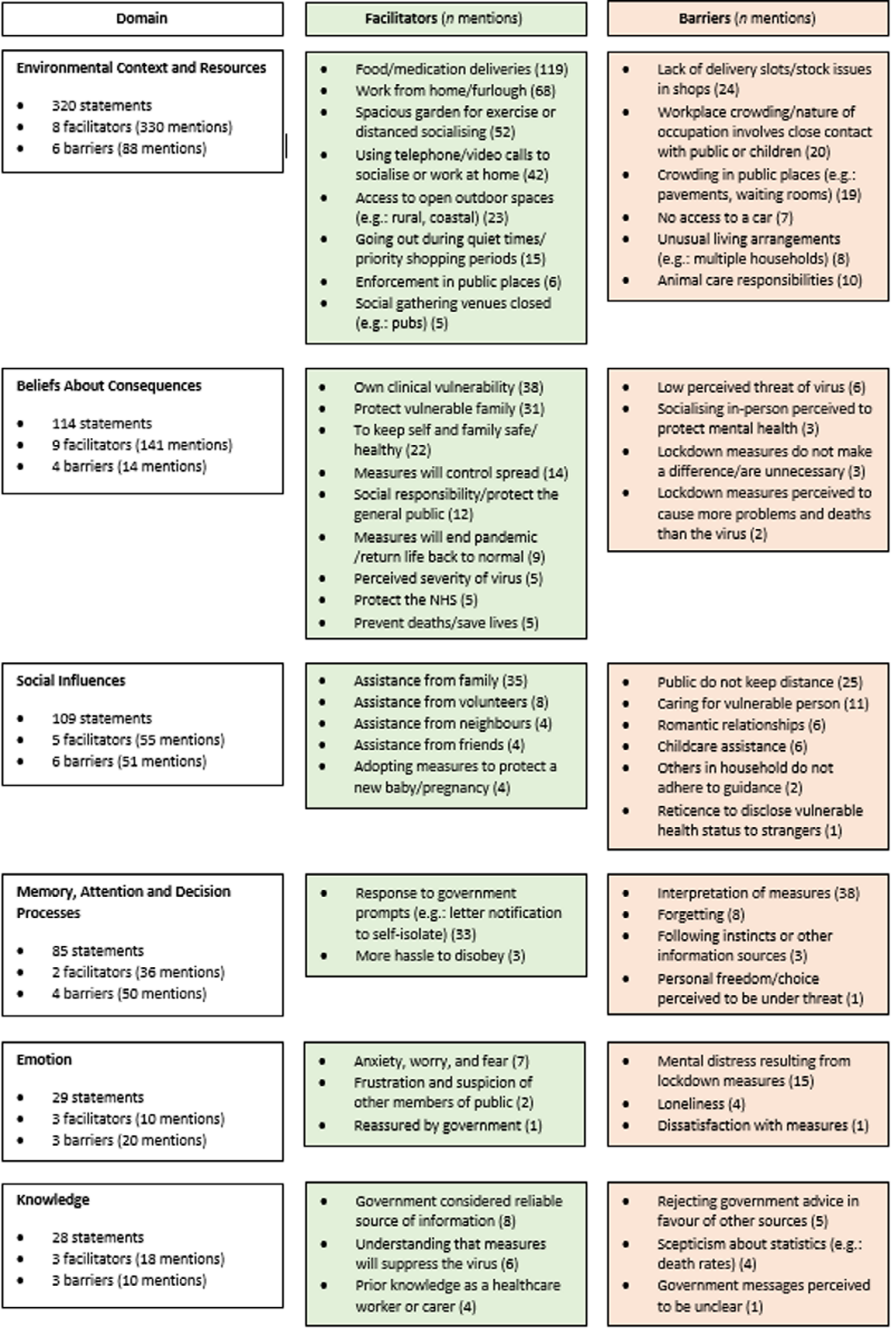
Facilitators and barriers from 498 statements presented by TDF domain

Of the 1,211 statements identified, a total of 498 (41.1%) were coded as containing a barrier or enabler; demographics of this sub‐sample are presented in Appendix E. These codes were mapped to relevant domains of the TDF framework. Thirteen determinants of behaviour were identified from the data, and 6 domains were considered important (illustrated in Table 2). A complete breakdown of determinants is depicted in Figure 1.

**Table 2:** Latent analysis summary

TDF domain	Description of domain	Exemplar quotes
Environmental Context and Resources	*Enablers*: Online deliveries; working from home; access to space for exercise; video conferencing; priority shopping periods; closure of social gathering places. *Barriers*: Workplace crowding; crowding in public places; lack of access to a car; stockpiling shortages; multiple households; animal care.	*Working full time from home, only leaving the house/property to exercise. Having shopping delivered… We live in a detached, rural property… so it is relatively easy to follow the guidelines*. (Participant 489) *I have gone shopping with someone I live with rather than going on my own, we do not own a car and therefore need extra hands to carry food shopping home*. (Participant 1521)
Beliefs About Consequences	*Enablers*: Perceptions that following the guidance would keep oneself and loved ones safe; high vulnerability to the virus; measures will control transmission and end the pandemic; social responsibility towards the NHS and the rest of society. *Barriers*: Low‐perceived threat of the virus, perceptions that socialising in‐person protects well‐being; beliefs that measures are ineffective; perception that lockdown measures cause more harm than the virus.	*Fed up? Yes, but believe it's necessary to protect ourselves and the NHS*. (Participant 1804) *I have not met friends but I will because we have all isolated and show no symptoms so don’t see what risk there is*. (Participant 648)
Social Influences	*Enablers*: Shopping assistance from family, neighbours, friends, or volunteers; adopting measures to shield babies or pregnant women. *Barriers*: Other members of the public not keeping physically distanced, caring responsibilities, romantic relationships; assistance with childcare; reticence to seek aid from volunteers.	*My shopping is brought by neighbour and family although this is not a lot as I am very much self‐sufficient*. (Participant 1934) *People come right next to me and I’ve had several arguments, they become immediately angry instead of stepping away, a lady has brushed me with her handbag*. (Participant 1529)
Memory, Attention and Decision Processes	*Enablers:* Government prompts; perceptions that it would be more difficult to breach government instructions than to follow them. *Barriers*: Interpretation of terms (e.g. ‘essential’); forgetting to wash hands or remain distanced; following instructions from other information sources; perceptions that autonomy is under threat.	*I am keeping a reasonable distance from others… not because I fear the virus… but because I respect their right to personal space and want to avoid unnecessary confrontation*. (Participant 2140) *I’m going on more than one walk per day because I smoke and cannot do that at home… I’ve forgotten to wash my hands after coming home a couple of times. (Participant 862)*
Emotion	*Enablers*: Feelings of anxiety, worry, and fear; vigilance around other members of the public; reassurance from the government. *Barriers*: Mental distress caused by staying at home and social distancing when living alone; loneliness; dissatisfaction with measures.	*Staying home getting bored now but… will be scared to go out before a vaccine is introduced*. (Participant 925) *We have not visited anyone outside of our household even though it has been an incredibly painful thing to do and has had a significant impact on my mental health*. (Participant 414)
Knowledge	*Enablers*: Government sources considered reliable; understanding why the measures are effective; personal/professional knowledge of hygiene measures. *Barriers*: Conflicting health guidance from other countries and public health organizations; scepticism about government statistics; perceived ambiguities in the instructions.	*I do these things because the advice given is sensible and relevant. This is a very dangerous virus*. (Participant 1903) *Not following the government's instructions at all: following good sense instructions from medical professionals* (Participant 1370)

#### Environmental context and resources (320 statements; 64.3% of 498)

Participants’ circumstances dictated whether their surroundings acted as an enabler or barrier. Living in rural areas, near green spaces, or coastline were cited as enablers to physical distancing, because daily exercise could be taken at a safe physical distance from others without travelling further afield. Spacious gardens enabled exercise at home, and provided a contained environment for physically distanced social interactions. Working from home and being furloughed were commonly mentioned enablers; access to video conferencing software enabled remote working ‘*rather than face to face’* (Participant 505), facilitated ‘*access [to] healthcare’* (Participant 797), and helped people keep in touch with friends and family. However, key workers were limited by their work environments. Physical distancing was described as ‘*impossible in a school setting’* (Participant 38) for teachers; bus and delivery drivers had ‘*issue[s]’* (Participant 231) staying away from customers; and supermarket staff felt ‘*more at risk [in work] than anywhere else’* (Participant 1565).

Access to resources such as medication and grocery delivery slots were important for staying at home during the lockdown; lack of access as a result of high demand and stock shortages due to stockpiling, meant many participants had to make several trips to different shops or risk ‘*run[ning] out of food’* (Participant 1637). This was further complicated by the absence of cars; those unable to carry groceries alone on foot would share the load with another person or make multiple trips. Crowding in shops, parks, and pavements was highlighted as a barrier to physical distancing by members of the public, however, some suggested the risks could be mitigated with marshals to limit numbers indoors, and priority shopping periods for key workers and clinically vulnerable people. Moreover, the shutdown of social gathering spaces like pubs was a reluctant facilitator for staying at home. A few participants described practical difficulties to staying at home if they lived between two households (e.g. in romantic relationships), or had care responsibilities for animals, such as horses and dogs.

We talk to family/friends on phone, Facebook, WhatsApp, we follow our church on [YouTube], We have weekly family time on Zoom. (Participant 812)

I don't have a car so can't carry a lot home, plus often the shops don't have what I need so I have to go back. (Participant 793)

#### Beliefs about consequences (114 statements; 22.9% of 498)

Participants held strong beliefs about the consequences of implementing the guidance. Many followed government measures ‘*in order to stay safe and keep others safe’* (Participant 1792) by avoiding infection. People with vulnerable relatives, or a vulnerable health status themselves, believed they were more susceptible, and the virus was a ‘*fatal threat’* (Participant 1038). Beyond personal and familial safety, participants described following the guidance as an act of good citizenship, because protecting vulnerable people across society was perceived as a ‘*responsible thing to do’* (Participant 365). Only a minority mentioned ‘*protect[ing] the NHS’* (Participant 1561) and ‘*sav[ing] lives’* (Participant 519) as a consequence, despite this being the tagline of the active public health campaign at the time. Participants were motivated to follow the government guidance because of the perceived effectiveness of the measures; there were salient beliefs that the measures would ‘*reduce the R and… ease lockdown quicker so life can return to normal’* (Participant 36).

In contrast, beliefs that the measures were unnecessary acted as a barrier, underlined by a low‐perceived threat of the virus. Participants were sceptical towards the time limit on daily exercise, and questioned the effectiveness of extra hygiene precautions (e.g. ‘*I wash my hands only if I have touched something outside, no need to otherwise’*; Participant 793). A common barrier for people living alone was the perceived negative consequences on mental health by remaining in isolation; one participant stated the lockdown was ‘*causing more problems than it’s solving’* (Participant 1735) in terms of mental distress, prompting them to socialize with friends. Participants who mentioned socializing to ease mental distress from lockdown were all unpartnered (i.e.: never married, separated, or widowed), and all but one were childless, suggesting those living alone may experience different barriers to following the instructions.

I do this to keep myself, family members and friends safe from Covid‐19. Following strict government guidelines for the good of the UK. I have Lupus, so I am scared of putting myself at risk… I don't want to die. (Participant 1709)

It would have been crazy to propose we move in together for the lockdown and neither of us wanted to… If I did not do this, I would be in a much worse mental state and very lonely. (Participant 842)

#### Social influences (109 statements; 21.9% of 498)

Clinically vulnerable participants were supported to stay‐at‐home by close family members from other households, such as parents or adult children. Assistance from neighbours, volunteer shoppers, or friends was less common, except among older participants (aged 55+). Participants who delivered these support mechanisms described their caring responsibilities as a barrier to following the guidance, since they needed to undertake more shopping trips and enter the home of the person they were caring for ‘*to deliver and unpack shopping as necessary and to take in meals’* (Participant 881). Additionally, one participant rejected the concept of receiving help from volunteers, because they did not want to disclose their health status to strangers.

Childcare responsibilities created both barriers and facilitators depending on the children’s age. Participants that recently had a baby were enabled to follow the guidance due to the extra precautions taken around maternity services. However, those with school‐age children described difficulties managing childcare around work commitments; a lack of ‘*respite from my children [and stress] about homeschooling’* (Participant 957) prompted them to seek cross‐household assistance from family members such as retired grandparents. Younger (aged 18–34), unmarried people in romantic relationships felt pressured to either move in together, or break the stay‐at‐home guidance to visit each other. Challenges with other household members not adhering were also described as a barrier. Unfortunately, participants across the sample frequently reported other members of the public as a barrier to physical distancing, due to a perceived lack of care. This led to confrontations and arguments with strangers when out exercising or shopping, while older participants expressed feelings of defeat (e.g. ‘*What can you do when people get too close?’*, Participant 906) and worry (e.g. ‘P*eople INVADE MY SPACE… and I am afraid to challenge them. I am Silver haired and concerned about them disrespecting me because of my perceived age.’*, Participant 699).

I try to stay 2 m away from everyone however this is not always possible in a small shop and most customers do not care about social distancing. (Participant 1565)

I am high risk/vulnerable but I have still been out to get supplies and to go to for walk just for my peace of mind and well‐being. Moreover, it’s not realistic to rely on someone else to get our supplies as you don't necessarily want people to know your health status. (Participant 1928)

#### Memory, attention, and decision processes (85 statements; 17.1% of 498)

In terms of deciding to adhere, participants prominently described government prompts as a catalyst for behaviour change; notably receiving a letter advising to self‐isolate on grounds of clinical vulnerability, and daily televised newscasts from the government. In addition to the health consequences outlined previously, participants mentioned adhering ‘*because the gov[ernment] advised’* (Participant 1391); that they were ‘*doing as asked’* (Participant 2029); or ‘*as I'm told’* (Participant 51). Others stated that they ‘*[would] not wait to be told’* (Participant 476), instead preferring to follow their instincts or adopt guidance from other health bodies that advocated for face coverings and additional hygiene precautions.

Forgetting was a common barrier for hand washing (e.g. ‘*Sometimes I don't always wash my hands when I come in’* Participant 596), physical distancing, and staying at home (e.g. ‘*…because we run out of food. If I planned properly, I wouldn't need to do this’* Participant 1637); however, these participants emphasized that lapses in memory were unintentional. In contrast, participants cited occasions where they broke the stay‐at‐home guidance deliberately by going out more than once a day because they decided it was not dangerous. These participants ‘*exercise[ed their] best judgement’* (Participant 304) to make decisions about their health. Similarly, participants described the term ‘essential’ in the guidance as having room for interpretation, using this as justification for trips ‘*just… to buy chocolate, lager’* (Participant 1720), to ‘*visit a local off license’* (Participant 2202), or to make ‘*purchases at hardware stores’* (Participant 304). Similar justifications were made about travelling for exercise in ‘*a different place’* (Participant 1506), or entering relatives’ households. Beyond interpretations of the guidance, participants argued that they should ‘*be free to make [their] own life decisions’* (Participant 1735), and considered the lockdown measures an ‘*infringement’* (Participant 793) on their capacity to do so. However, they also conceded that it was more troublesome to become involved in confrontations, than to abide physical distancing: ‘*it is easier to follow rules than to disobey them’* (Participant 119).

Some of the instructions are open to interpretation and some other people may not agree… I may go on a long walk or cycle ride… just as I would have done before Covid, but I will maintain distances… Also, I have visited a shop to buy non‐food goods, which some people seem to think is wrong. (Participant 1506)

#### Emotion (29 statements; 5.8% of 498)

A range of emotional determinants impacted participants’ behaviour. Negative emotions were prevalent among participants’ statements, which characterized both barriers and facilitators. Feelings of ‘*anxiety’* (Participant 593), worry, and fear of persecution (e.g. ‘*I am afraid that the police will arrest me and destroy my life if I go outside at all’*, Participant 1249) enabled people to stay‐at‐home; some patients were disinclined to go outside due to fear of the virus being ‘*just outside my front door’* (Participant 2213). Frustration and suspicion of others encouraged vigilance when distancing out in public. By contrast, some people felt so distressed by living on their own during the lockdown that they broke regulations to socialize with others to ‘*[help them] not to struggle’* (Participant 139). Similarly, going out several times a day was described as essential for maintaining well‐being and preventing deterioration. This was common among those who had existing mental health difficulties; a participant with a history of substance use said they ‘*get anxious [being] indoors too long so I go [out] several times a day for short walks’* (Participant 1172). Emotional resilience was characterized as an enabler; although one participant reported that they would follow the guidelines until they could not ‘*stand it any more’* (Participant 441), suggesting the emotional toll of the guidelines had a deleterious impact on people’s motivation to implement the guidelines. Finally, emotional reactions towards the source of the guidance were both enablers and barriers, based on whether the government guidance was considered reassuring (e.g. ‘*I am doing everything that the government advise me to do, I have every faith in them*.*’*, Participant 2075) or frustrating (e.g. ‘*I am also not going out as much, solely because everywhere has been forced to shut (by the heavy hand of the nanny state, supported by a cowardly population…)’,* Participant 2140).

I do not leave my house, anxiety stops me… even when it comes to essential shopping, i have been to the shops twice i think since lock down started. (Participant 593)

#### Knowledge (28 statements; 5.6% of 498)

Participants reported that they were committed to following advice from the government because they were told it was important to reduce the R rate and suppress the spread of the virus. Some described the government communications as ‘*sensible and reassuring’* (Participant 337), and sourced their information from news websites, daily briefings, and mobile alerts. Some participants relied on their personal knowledge of preventative measures (e.g. ‘*following aseptic technique’*, Participant 1308) from their professional roles in healthcare in addition to the government advice about hand hygiene. However, a minority felt distrust towards messages about COVID‐19 from the government, scepticism towards infection and death rate statistics, and rejected mainstream media; these participants described confusion about perceived ‘*mixed messages’* (Participant 305) coming from Westminster and devolved governments (e.g. Scotland) at the time, especially about whether additional measures such as face coverings were necessary. These participants were all aged between 35 and 62, and three‐quarters of them were from a lower socioeconomic background. Other sources were perceived to be more reliable than the government, such as foreign news outlets, trusted medical professionals, and the World Health Organization; as such, these participants followed their advice instead.

I'm doing this because of the information we have been given by Professor Whitty and his team. (Participant 1120)

## Discussion

This paper aimed to explore how members of the UK public described their behaviour in relation to government COVID‐19 guidance, and identify salient determinants of COVID‐related behaviours. Staying at home was the most commonly described guideline measure (70% of statements), followed by hand washing (44%), and physical distancing (40%); as staying at home represented the most extreme and controversial change to daily life, it is unsurprising this was an important issue.

Thirteen determinants of behaviour were identified using the TDF framework, 6 of which were considered important based on the volume of statements relating to each domain. Environmental Context and Resources was the most prominent determinant that enabled staying at home and physical distancing, due to the availability of cars, uncrowded spaces, video conferencing software, and grocery delivery slots. Conversely, lack of access to such resources and environmental limitations in workplaces, public spaces, and households were problematic. This corroborates existing findings highlighting socioeconomic inequities that cause barriers to public adherence (Benham et al., 2021; Hills & Eraso, 2021). Our findings complement research suggesting green spaces, particularly in urban areas, are subject to bottlenecks and overcrowding which discourages access due to safety concerns (Burnett, Olsen, Nicholls, & Mitchell, 2021; Shoari, Ezzati, Baumgartner, Malacarne, & Fecht, 2020); such barriers compound for people on low incomes, from minority ethnic groups, or living in areas of deprivation (Cronin‐de‐Chavez, Islam, & McEachan, 2019). Determinants relating to Environmental Context and Resources domain overlapped with the Social Influences domain; support from family and neighbours to provide groceries and medication enabled vulnerable individuals to stay‐at‐home, contrasting with a Belgian survey that found no association between social support and stay‐at‐home behaviour (Beeckman et al., 2020). However, members of the public were a source of frustration and conflict for physical distancing during exercise and shopping, which supports existing findings that successful distancing is contingent upon mutual cooperation (Gouin et al., 2021).

Another prominent determinant was Beliefs About Consequences; participants were motivated to adhere if they believed they were protecting themselves or a vulnerable loved one, which is a powerful motivator (Sturman et al., 2020). Participants felt a sense of duty to protect the country and NHS, which echoes findings from other countries about ‘civic’ duties and social responsibility (Coroiu et al., 2020; Hassan et al., 2021). In contrast, negative beliefs about the guidance being excessive or ineffective were reasons for non‐adherence, consistent with findings about the adoption of protective measures such as face coverings (Taylor & Asmundson, 2021). Furthermore, a meta‐analysis of government interventions found that public support for such policies is sensitive to their perceived effectiveness, suggesting that support for COVID‐19 instructions could be increased by sufficiently and clearly communicating the effectiveness of the measures (Reynolds, Stautz, Pilling, van der Linden, & Marteau, 2020). There was thematic convergence between Beliefs about Consequences and the Knowledge domain; information about COVID‐19 obtained from government sources facilitated adherence (Gao et al., 2020; Vo et al., 2020), but competing information sources and lack of trust diluted the main message (Fancourt, Steptoe, & Wright, 2020).

Memory, Attention and Decision Processes about guideline behaviours were influenced by forgetting (particularly handwashing), appraisals, and interpretations of government terminology. Considering 70% of statements included exemption‐related terms such as ‘essential’, these concepts are likely to have informed public behaviour (Smith et al., 2020). While the frequency of these terms was mostly due to participants describing essential travel or shopping, the mentions of ambiguity are consistent with research where differing interpretations were used to justify non‐essential violations (Williams et al., 2020). Emotional barriers to staying at home and physical distancing were prominent, corroborating findings about the psychological toll of adherence to lockdowns (Margraf et al., 2020). However, emotional motivations had mixed impacts on adherence; feelings of fear and anxiety encouraged staying at home for some, while others felt unable to keep distanced from loved ones due to mental distress. This inconsistency was reported in another UK sample, where people who expressed more fear of COVID‐19 made more non‐essential trips (Kooistra et al., 2020), and calls into question the roles of ‘functional fear’ and threat appraisal on adherence (Harper, Satchell, Fido, & Latzman, 2020; Lithopoulos et al., 2021). Although fear‐based messaging can be an effective strategy to influence attitudes, intentions, and one‐time behaviours (e.g. vaccination) provided it is bolstered by efficacy messaging, it is less effective at changing the kinds of recurring behaviours contained in the government guidance (Tannenbaum et al., 2015); indeed, the effect of fear on COVID‐19‐related compliance is small when self‐efficacy is high, suggesting self‐efficacy may be a more appropriate target for intervention (Jørgensen, Bor, & Petersen, 2021). There was further convergence between the Emotion, Beliefs About Consequences, and Memory, Attention and Decision Processes domains; beliefs about negative emotional consequences of long‐term adherence to the government instructions prompted people living alone to disengage with the stay‐at‐home guidance.

## Implications

These findings detail the experiences of a representative sample of UK adults during the first national lockdown. In the context of the wider sample these data were captured from, challenges to guideline adherence were common and varied, particularly in relation to adjustments to daily routines and impacts on mental and physical health (Keyworth et al., 2021). The sample had few physical and social opportunities to adhere (Armitage et al., 2021), which complements our findings that environmental context and social influences were the two most prominent determinants of behaviour. Although most of the samples were capable and motivated to follow government guidance, their behaviour was restricted by problematic environments, a lack of resources, and limited mutual support. Since these domains are least amenable to individual behaviour change due to the structural nature of environment and resource barriers, behaviour change interventions may not be an appropriate approach to support members of the public. Instead, policy makers and governmental health bodies should be targeted to ensure future initiatives to promote adherence account for inequities exacerbated by government measures (Chater et al., 2021; Michie et al., 2020). Future work could utilize the Behaviour Change Wheel to identify appropriate intervention functions and policy categories to guide the design process of such initiatives (Michie et al., 2014).

A key principle for COVID‐19 public health campaigns was to ‘make it possible’ by providing support to those affected by the measures, in the form of redistributive policies including income protection, food provision, and access to education (Bonell et al., 2020). Although some measures such as the furlough scheme and priority delivery slots were introduced early on, few continued long term. For example, volunteer support networks appeared during the initial lockdown to coordinate deliveries to vulnerable people (Smith et al., 2020) but were not reinstated during subsequent lockdowns; future initiatives should aim to financially support community‐led networks to support the vulnerable and increase mutual caring behaviours (Drury, Carter, Ntontis, & Guven, 2021). Evidence from countries such as Vietnam where cases remained low throughout the pandemic attribute partial success of their measures to the provision of essential supplies and services by the government to facilitate stay‐at‐home and distancing measures (Vo et al., 2020).

Determinants within domains such as Beliefs About Consequences, Knowledge, Emotions, and Memory, Attention and Decision Processes may provide some opportunities to optimize the public’s capabilities and motivations to adhere. Health messaging should emphasize the usefulness and effectiveness of measures, to justify the personal sacrifices demanded of the public and increase policy support (Gouin et al., 2021; Reynolds et al., 2020). Messaging should go beyond knowledge provision by emphasizing the pro‐social benefits of adherence, to elicit supporting emotions, such as connectedness and hope to motivate those who are health‐literate (Berg‐Beckhoff, Dalgaard Guldager, Tanggaard Andersen, Stock, & Smith Jervelund, 2021; Hills & Eraso, 2021). The promotion of prosocial norms and a sense of collective identity in health messages can help to modify self‐centred motivations (Bonell et al., 2020). The introduction of ‘support bubble’ systems between households in subsequent lockdowns may have eased the emotional burden of staying at home while suppressing transmission (Leng et al., 2021). Messages that reduce fear and include instructions on how to bolster well‐being, self‐efficacy, and emotional regulation without deviating from the guidance may help tackle the emotional toll of lockdown measures (Armitage et al., 2021; Jørgensen et al., 2021).

## Limitations

The data were collected during the original lockdown; since then, government guidance changed multiple times (e.g. local lockdowns, Tier systems, ‘Stay Alert’) (Nartowski et al., 2020). New, more important determinants may have emerged in the wake of changing public mindset about more recent guidance. Self‐reported measures of guideline adherence are overestimated (Mieth et al., 2021), and social desirability biases may mean instances of guideline non‐adherence were not described. We did not separate self‐isolation or quarantine behaviours, which are the poorest adhered to and in greatest need of intervention support (Smith et al., 2020). Given the removal of lockdown restrictions and adoption of the Track and Trace system, self‐isolation in response to contact notifications and quarantine measures following international travel are likely to be the most salient behaviours to control the spread of COVID‐19 in future (Cevik, Baral, Crozier, & Cassell, 2021). While our sample was intended to be nationally representative, there was an over‐representation of older, White people of a higher socioeconomic status. Since the data were collected online, it is likely that additional determinants from marginalized groups, such as those with limited access to technology or limited literacy skills, were missing from our data (Braun et al., 2020). We used an open‐ended question to gather data. Although this allowed us to code spontaneous themes from the data, tailoring questionnaire prompts to TDF domains could have provided more data about overlooked domains.

### Conclusions

This study adds to the body of literature attempting to catalogue determinants of compliance to government COVID‐19 guidance, and provides insight into the ways the British public describe their adherence to these measures. We observed six domains that influenced adherence: Environmental Context and Resources; Beliefs about Consequences; Social Influences; Memory, Attention and Decision Processes; Emotion; and Knowledge. Despite extensive structural obstacles, the majority of the British public were able to follow government COVID‐19‐related instructions provided they had sufficient resources, social support, and positive perceptions about the effectiveness of the measures. Ambiguities surrounding key terminology in the guidance left room for interpretation, which may have contributed to non‐adherence. This paper outlines important challenges to be addressed by policymakers and government health bodies to facilitate adherence to future government health messages.

## Conflict of Interests

JZL reports grants from NIHR Greater Manchester Patient Safety Translational Research Centre; CJA was supported by NIHR Manchester Biomedical Research Centre.

## Author contribution

**Jessica Zita Leather:** Data curation; Formal analysis; Project administration; Visualization; Writing – original draft. **Chris Keyworth:** Conceptualization; Formal analysis; Funding acquisition; Project administration; Supervision; Writing – review & editing. **Tracy Epton:** Conceptualization; Funding acquisition; Writing – review & editing. **Joanna Goldthorpe:** Writing – review & editing. **Fiona Ulph:** Writing – review & editing. **Christopher J Armitage:** Conceptualization; Funding acquisition; Supervision; Writing – review & editing.

## Funding information

The study was funded by the Manchester Centre for Health Psychology at the University of Manchester, and supported by the National Institute for Health Research (NIHR) Manchester Biomedical Research Centre, and supported by the NIHR Greater Manchester Patient Safety Translational Research Centre (award number: PSTRC‐2016‐003). The views expressed are those of the author(s) and not necessarily those of the NIHR or the Department of Health and Social Care.

## Data Availability

Data available on request from the authors.

## APPENDIX C

### Table demonstrating how responses queried by Coder 1 as not fitting TDF domains were resolved through discussion with Coder 2

**Table:**

Queried Quotes	Coder 2 Domain	Final agreed domains
Because of my age I do not go to the shops or outside of the home. I take my dog once a day literally round the block to enable him to do his business. *The only rule I broke was to visit my mother on her 95th Birthday* but we both stayed in her garden and were at least 2 metres apart at all times. I live with m6 daughter, son‐in‐law and teenager granddaughter but I only have my main Meal with them and most other times, I stay in my self‐contained apartment	Behavioural regulation	Behavioural Regulation; Environmental context and resources; Social influences
I’m following the instructions *until I can’t stand it any more*	Emotion	Emotion
I’m disabled and *its just a normal day for me*,,,the only difference is my regular hospital and doctor appointments have been cancelled	Behavioural regulation	Behavioural regulation; Memory, attention, and decision processes
Following the government guidelines and only going out food shopping once or twice a week for food shopping. However, I am *going inside my mother's house (she is 93) to deliver and unpack shopping as necessary and to take in meals I have cooked for her*	Memory, attention and decision processes	Memory, attention, and decision processes, Social influences
I am currently 8 months pregnant so I am adhering to the guidelines as much as possible. In the previous 6 weeks I have spent 95% of my time at home, leaving only once per week to do the essential food shop or to go to our small farm to help my husband feed calves. I have stopped any visitors from coming to our home, our son has only been in our home or in our car to the farm and we have upped hand washing etc My husband however had to continue to work for the first 3 weeks as his employer was not ordered to close. Although they tried to adhere to the guidelines, he was continually coming into contact with many others. He has now been furloughed for 3 weeks initially however again as we have a small farm he has to come in to contact at agri supply stores, vets etc. *We have found that rurally people are not as inclined to adhere to social distancing*	Environmental context	Environmental context, Social influences
I am doing everything that the government advise me to do*, I have every faith in them*. I only leave the house and garden to walk and get exercise once daily, stay socially distant from people I come across. Wash my hands regularly, and keep in contact with family and friends via telephone and ipad	Optimism	Optimism, Emotion, Memory, attention, and decision processes
I am doing my best to stay‐at‐home, but *have socialised in a very minor way in order to keep my sanity*. I have seen one friend occasionally	Beliefs about consequences	Beliefs about consequences, Emotion
I am *following the Scottish Governments' instructions*. I live alone and have compromised mobility. *My groceries are delivered*. I have gone out once for a G.P. appointment ‐ not Covid‐related. I drove to the G.P. surgery and back and came into contact with no‐one other than my G.P. I wore a mask when out. Otherwise, I wash my hands where and when appropriate. I do these things because *the advice given is sensible and relevant*. This is a very dangerous virus. I make contact with others by *telephone and the Internet*	Beliefs about consequences	Beliefs about consequences, Knowledge, Memory, attention, and decision processes, Environmental context and resources
I am following them all, but *some of the instructions are open to interpretation and some other people may not agree with my interpretation*. For example, some days I may go on a long walk or cycle ride (perhaps 2 or 3 hours), just as I would have done before Covid, but I will maintain distances etc. Also, I may drive a few miles to a different place for a walk. Also, I have visited a shop to buy non‐food goods, which some people seem to think is wrong	Memory, attention, and decision processes	Memory, attention, and decision processes
I am staying at home unless I need shopping. Usually once a week. I speak to my neighbours at a safe distance. I have arranged to have some meals delivered so that I don't need so much shopping. I wear mask and gloves in shops, If I have worn the gloves, they get washed that night. I wash and sanitize my hands. If my sister has anything for each other. She leaves it outside her house and I go there, collect it and leave my bag. My friends dog died and I would normally have given her a cuddle. I felt dreadful because I could not go to her. *I consider myself one of the lucky ones because I have a garden and lots of indoor interests to keep me occupied*. When I am shopping, I ask my friends if they need anything and get it for them to save them going out. Most of them do not drive or have a nearby bus service. One of them the bus service has been stopped. However, I do not take anyone in my car. No one has been in my car since the lockdown only a dog.	Environmental context	Environmental context and resources, Emotion, Social influences
I am staying in except for food, medicine and one form of exercise per day. I am social distancing when out. *I’m not wearing a mask as government have not made this official advice*.	Memory, attention, and decision processes	Memory, attention, and decision processes
I live alone and i now work from home in both of my jobs and socialize with friends online only. I was in a kind of non‐committal sexual relationship with someone who lives alone and a 2‐ walk from my house and we spent the weekend together before the lockdown and were together when it was announced. It would have been crazy to propose we move in together for the lockdown and neither of us wanted to so that so we agreed to only have contact with one another but we do that across two households. *I think that if i did not do this i would be in a much worse mental state and very lonely as my family do not live in the same city and my friends are not in a situation where i could move in with them, plus i want to security of being in my own home, however I'm aware it is a 'cheat', it is not allowed so I'm scared people at my work will ask me about it because i am a bad liar*. In terms of exercise i go for runs and sometimes longer walks for a few hours, this is also a good way of pas	Beliefs about consequences	Beliefs about consequences, Emotion, Social influences
Only travelling to work one day a fortnight (I’m a teacher on a rota). Not having physical contact with anyone apart from my immediate family. Only using the car to go to work occasionally and drive once a week to buy food. Washing hands more often. Remaining as far as physically possible from people; we spend quite a bit of time chatting to friends and neighbours from opposite ends of garden paths though. Haven’t been in anyone else’s house for 6 weeks. I do leave the house more than once a day to exercise though; *I go for a run in the morning then walk the dog in the afternoon because if I don’t run then I’ll go mad and the dog won’t run with me*.	Emotion	Emotion
Washing hands after shopping / being out. Washing hands more regularly. Keeping at least two metres away from people where possible. Only going out for exercise and shopping. *This advice is given to the government by experts* and therefore I am following it.	Memory, attention and decision processes	Memory, attention, and decision processes
Trying to follow them as closely as possible but *making occasional trips to carry out jobs for a vulnerable parent*	Memory, attention and decision processes	Memory, attention and decision processes
Stay‐at‐home all the time, keep a distance of 2 metres from anyone who delivers anything, wash hands for 2 minutes frequently, get *food and medicines delivered*, … it's been more difficult to get my *19‐year‐old son* to always follow the instructions at first, he was still seeing his girlfriend at weekends, but I explained to him that they had to make a decision and *how dangerous it was*. it took some time for them both to take it seriously but now they do. *I think the mixed messages from government didn't help at that stage*. now they stay apart	Environmental context	Environmental context and resources, Knowledge, Memory, attention, and decision processes, Social influences
Following all instructions except *possibly not interpreting "essential" correctly and exercising my best judgement,* for example, purchases at hardware stores	Memory, attention and decision processes?	Memory, attention, and decision processes
Because of my age I do not go to the shops or outside of the home. I take my dog once a day literally round the block to enable him to do his business. *The only rule I broke was to visit my mother on her 95th Birthday* but we both stayed *in her garden* and were at least 2 metres apart at all times. I live with m6 daughter, son‐in‐law and teenager granddaughter but I only have my main Meal with them and most other times, I stay in my self‐contained apartment	behavioural regulation	Behavioural regulation, Social influences, Environmental context and resources

## APPENDIX D

### Example of thematic codes mapped to quotes within a single domain, illustrating how overlap between domains was labelled

**Table:**

Beliefs About Consequences			
Quote	Barrier	Facilitator	Domain Overlap
I am staying at home, and only leaving for essential food shops. *I am doing this because it is important to reduce the spread of the virus as much as possible*. I am following this at all times. I am keeping 2m apart from others when there is a need to be close at all. I am also regularly washing hands. *All this is to reduce the R and to try and ease lockdown quicker so life can return to normal sooner*		Belief that lockdown will reduce spread; Return life to normal	Knowledge (Lockdown/reduced movement will suppress virus spread and reduce R, reducing need for lockdown)
I am complying with the govt restrictions *whilst not agreeing they are entirely necessary*. I wash my hands frequently, always on returning from going outside, I keep 2 metres distance from others, I limit my excursions outside the home to once per week by car to nearest supermarket, plus daily exercise from the home door for up to 2 hours. I have not seen a member of my family or made any journey other than to supermarket since lockdown began.	Measures not believed to be necessary.		None
I go out every day for an exercise walk from home, keeping at least 2 metres from anyone I see. I quarantine groceries and wash my hands more than usual *(although I suspect I might occasionally forget)*. All my family visits and social events have ceased, *except online*. I am a volunteer on the Responders system and do visit pharmacies to pick up prescriptions for others, but I observe social distancing. *I think its vital for all to comply to save lives and I am very accepting of the restrictions for that reason*.		Belief that compliance will save lives Online socialization (Environmental Context and Resources) Forgetting (Memory, Attention, and Decision Processes)	Memory, attention, and decision processes (suspected forgetting)
Not going out unless essential, but sometimes going out more than once a day for walk. *I live in a rural area so do not see people, therefore I do not see it as a risk*	Area perceived to be low risk.	Access to green/open space (Environmental context and resources)	Environmental context and resources (access to and perceptions of risk in different areas)

## APPENDIX E

### Demographic information

**Table:**

Variable	Total *n* (2252)	Total % (2252)	Sub‐sample *n* (498)	Sub‐sample % (498)
Age	(M = 50.34, SD = 17.02)		(M = 52.50, SD = 17.49)	
18–24	165	7.3	27	5.4
25–34	316	14.0	73	14.7
35–44	396	17.6	78	15.7
45–54	398	17.7	73	14.7
55+	977	43.4	247	49.5
Gender				
Male	1018	45.2	167	33.5
Female	1234	54.8	331	66.5
Ethnicity				
White British	2001	88.9	448	90.0
Irish	32	1.4	7	1.4
Gypsy/Irish Traveller	2	0.1	1	0.2
Other White background	83	3.7	16	3.2
White and Black Caribbean	9	0.4	1	0.2
White and Black African	6	0.3	0	0.0
White and Asian	7	0.3	0	0.0
Other mixed/multiple ethnic background	12	0.5	3	0.6
Indian	26	1.2	5	1.0
Pakistani	7	0.3	2	0.4
Bangladeshi	5	0.2	1	0.2
Chinese	12	0.5	2	0.4
Other Asian background	4	0.2	1	0.2
African	8	0.4	1	0.2
Caribbean	6	0.3	2	0.4
Other Black/African/Caribbean background	1	< 0.1	0	0.0
Arab	1	< 0.1	0	0.0
Any other ethnic group	11	0.5	4	.8
Prefer not to say	19	0.8	4	.8
Country of residence				
England	1875	83.2	403	80.9
Wales	107	4.8	28	5.6
Scotland	209	9.3	52	10.4
Northern Ireland	61	2.7	15	3.0
Social Grade				
Upper	1419	63.0	312	62.7
Lower	833	37.0	186	37.3
Work status				
Full time	892	39.6	159	31.9
Part time	324	14.4	78	15.7
Full time student	87	3.9	11	2.2
Retired	618	27.4	176	35.3
Unemployed	89	4.0	13	2.6
Not working/other	242	10.7	61	12.3
Marital status				
Married/Partnered	1089	48.6	236	47.8
Living as married	290	13.0	57	11.5
Separated/Divorced	206	9.2	51	10.3
Widowed	87	3.9	39	7.9
Never married	566	25.3	111	22.5
Parent/guardianship				
No	919	40.8	194	39.0
Yes	1333	59.2	304	61.0
Child aged < 4	174	7.7	42	8.4
Child aged 5–11	271	12.0	52	10.4
Child aged 12–16	203	9.0	40	8.0
Child aged 17–18	81	3.6	15	3.0
Child aged > 18	877	38.9	209	42.0
Children living in household				
None	1622	72.0	365	73.3
1 child	279	12.4	67	13.5
2 children	229	10.2	45	9.0
3+ children	83	3.7	15	3.0

NB: Of the total sample 32 (1.4%) did not provide any demographic details; 46 (2%) did not provide marital status, and 39 (1.7%) did not answer about children. Of the sub‐sample, 4 (0.8%) did not provide marital status and 6 (1.2%) did not answer about children.
